# Novel Tripodal Polyamine Tris-Pyrene: DNA/RNA Binding and Photodynamic Antiproliferative Activity

**DOI:** 10.3390/pharmaceutics15092197

**Published:** 2023-08-25

**Authors:** Marta Jurković, Marijana Radić Stojković, Ksenija Božinović, Davor Nestić, Dragomira Majhen, Estefanía Delgado-Pinar, Mario Inclán, Enrique García-España, Ivo Piantanida

**Affiliations:** 1 Division of Organic Chemistry and Biochemistry, Ruđer Bošković Institute, Bijenička Cesta 54, 10000 Zagreb, Croatia; marta.koscak@irb.hr (M.J.); mradic@irb.hr (M.R.S.); 2 Division of Molecular Biology, Ruđer Bošković Institute, Bijenička Cesta 54, 10000 Zagreb, Croatia; ksenija.bozinovic@irb.hr (K.B.); davor.nestic@irb.hr (D.N.); dragomira.majhen@irb.hr (D.M.); 3 Department of Inorganic Chemistry, Institute for Molecular Science, University of Valencia, Catedratico Jose Beltran 2, 46980 Paterna, Spain; estefania.delgado@uv.es (E.D.-P.); mario.inclan@uv.es (M.I.); 4 Escuela Superior de Ingeniería, Ciencia y Tecnología, Universidad Internacional de Valencia (VIU), 46002 Valencia, Spain

**Keywords:** polyamines, pyrene, DNA and RNA binding, photodynamic therapy, antiproliferative activity

## Abstract

A novel tri-pyrene polyamine (**TAL3PYR**) bearing net five positive charges at biorelevant conditions revealed strong intramolecular interactions in aqueous medium between pyrenes, characterised by pronounced excimer fluorescence. A novel compound revealed strong binding to ds-DNA and ds-RNA, along with pronounced thermal stabilisation of DNA/RNA and extensive changes in DNA/RNA structure, as evidenced by circular dichroism. New dye caused pronounced ds-DNA or ds-RNA condensation, which was attributed to a combination of electrostatic interactions between 5+ charge of dye and negatively charged polynucleotide backbone, accompanied by aromatic and hydrophobic interactions of pyrenes within polynucleotide grooves. New dye also showed intriguing antiproliferative activity, strongly enhanced upon photo-induced activation of pyrenes, and is thus a promising lead compound for theranostic applications on ds-RNA or ds-DNA targets, applicable as a new strategy in cancer and gene therapy.

## 1. Introduction

Nature intrinsic polyamines (e.g., spermine, spermidine, and putrescine) are quite common and often essential participants in many biological processes, such as cell life and proliferation [1,2,3,4]. Many of these processes are associated with polyamine interaction with DNA and the ability to alter DNA conformation through condensation and aggregation [5,6].

Polyamines dominantly base their non-covalent interaction with nucleic acids on electrostatic attraction between the positively charged protonated amines and the negatively charged phosphates of DNA or RNA [7]. For instance, a small change in the pH of a solution can reversibly influence protonation of amine and in this way control the interaction with DNA [8].

**Scheme 1:**
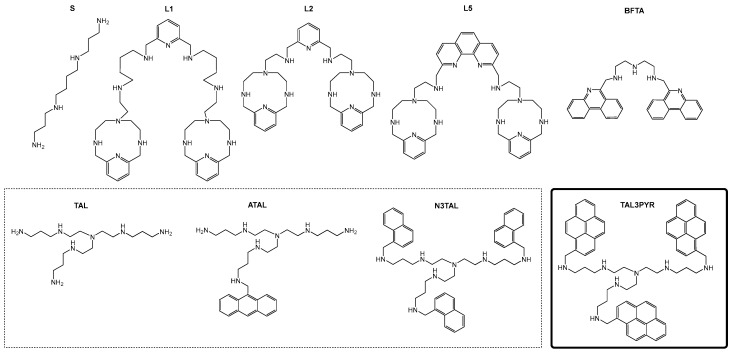
Structures of spermine (**S**), previously studied **L1**–**L5** [16,17,18], **BFTA** [19], starting compound **TAL**, and previously prepared analogues **ATAL** and **N3TAL** [20], and the here-studied **TAL3PYR**. Note that protonation of aliphatic amines allows for pH-control of positive charge and consequent electrostatic interactions with negatively charged DNA or RNA backbone.

For example, spermine (S in Scheme 1), depending on the base composition of the ds-DNA, can show either negligible base-pair selectivity [9,10,11] or GC selectivity [12,13,14]. In addition, polyamines preferentially interact with bent adenine tracts in double-stranded DNA [2], or some natural polyamines induce refolding of the B-secondary DNA structure into the A- or Z-structures [15], or they can induce DNA aggregation [6].

Inspired by the multitude of finely regulated interactions with DNA/RNA, our groups over the last decade designed and studied novel synthetic open-chain polyamine ligands able to bind selectively RNA over DNA [16]. However, the first family of ligands based on enlarged tris(2-aminoethyl) moieties functionalised with aromatic units, as poor cellular intake showed no significant in vitro activity in cancer cell cultures; non-efficient transfer through cellular membranes was attributed to too high positive charge in physiological conditions. We designed more hydrophobic aryl-linked (pyridine and phenanthroline) bis-polyaza pyridinophane ligands (**L2** and **L5** in Scheme 1) [17], which showed selectivity for ds-RNA (poly A–poly U) over ds-DNA (poly dA–poly dT), attributed to the differences in the steric properties of the binding sites of these polynucleotides [13]. Interestingly, the much lower ds-RNA over ds-DNA selectivity observed for **L5** in comparison to **L2** was ascribed to the ability of phenanthroline to intercalate between the base pairs of the polynucleotides. These compounds condensed GC-DNA into ψ-DNA [18]. 

Further, we also studied DNA-intercalating moieties conjugated to polyamines, noticing that the bis-phenanthridine-analogue (**BFTA**, Scheme 1) had intriguing pH-controlled spectrophotometrical properties, as well as pH-modulated interactions with DNA/RNA [19]. In view of these promising results, we looked for a larger condensed polyaromatic moiety. Pyrene was chosen as it is one of the most widely investigated fluorophores and is highly sensitive to its microenvironment. Moreover, pyrene is particularly interesting due to its excimer and exciplex formation characterised by strong bathochromically shifted emission [21], a very useful feature for the selective sensing of various targets. For these reasons pyrene-based probes were used for sensing various DNA/RNA/proteins [22,23], including theranostic applications [24]. Moreover, pyrene-peptide conjugates demonstrated fine sensing of various nucleic acid sequences [25,26]. Pyrene-analogues were also studied as anticancer agents/chemotherapeutics [27,28]. For this, research was particularly intriguing concerning the property of pyrene to produce singlet oxygen upon irradiation [21], which could eventually lead to photo-induced DNA cleavage or bioactivity [28,29]. Indeed, taking into account recent advancements in Two-Photon-Absorption (TPA) techniques applied for photodynamic therapy (PDT) [30,31], pyrenes can now be applied as very efficient photosensitisers [29,30,31,32]. 

Here we present the results of a study the interaction of **TAL3PYR** with double-stranded polynucleotides characterised by significantly different secondary structures (Table S1 in SI): (i) ctDNA, which presents a classical B-helix and balanced AT-GC base pair composition [33,34], (ii) dGdC-DNA, which also forms classical B-helix but with a minor groove that is sterically hindered by amino groups of guanine, (iii) dAdT-DNA, which forms a classical B-helix minor groove, ideal for small molecule binding [35], and (iv) rArU-RNA (ds-RNA), characterized by A-helical structure of wide and shallow minor groove and deep and narrow major groove. The latter is available for small molecule binding [33,35].

## 2. Materials and Methods

### 2.1. General Synthesis

Solvents were distilled from appropriate drying agents shortly before use. TLC was carried out on DC-plastikfolien Kieselgel 60 F254 and preparative thick layer (2 mm) chromatography was conducted on Merck 60 F254 plates (Merck KGaA, Darmstadt, Germany). (Merck, Merck KGaA, Darmstadt, Germany). NMR spectra were recorded on AV600 and AV300 MHz spectrometers (Bruker BioSpin GmbH, Rheinstetten, Germany), operating at 150.92 or 75.47 MHz for ^13^C and 600.13 or 300.13 MHz for ^1^H nuclei, using DMSO-*d*_6_ as the internal standard (labels in the spectra: Pyr = pyrene). Mass spectrometry was performed with an Agilent 6410 Triple Quad mass spectrometer (Agilent Technologies, Santa Clara, CA, USA) and high-resolution mass spectra (HRMS) were obtained using a Q-Tof2 hybrid quadrupole time-of-flight mass spectrometer (Micromass, Cary, NC, USA).

The starting tripodal polyamine *N,N’-{2-Bis[2-(3-aminopropylamino)ethyl]aminoethyl}-1,3-propanediamine* (**TAL**) was prepared following a synthetic strategy as previously described [20].

*N-1-Pyrenylmethyl-N’-(2-bis{2-[3-aminopropylamino]ethyl}-aminoethyl)-1,3-propanediamine heptahydrochloride* (**TAL3PYR·7HCl**).

A solution of 1-pyrenecarboxaldehyde (957 mg, 4.16 mmol) in 150 mL of dichloromethane was added dropwise to a solution of N,N′-{2-Bis[2-(3-aminopropylamino)ethyl]aminoethyl}1,3-propanediamine (**TAL**) (440 mg, 1.38 mmol) in 100 mL of dry ethanol under argon. The resulting solution was stirred for 24 h at room temperature and protected from the light. NaBH_4_ (1.7 g, 45 mmol) was then added, and the solution was stirred for 3 h. The solvent was then removed under reduced pressure and the residue obtained was treated with water and extracted with dichloromethane (3 × 50 mL). The organic phases were collected and dried under reduced pressure. The resulting oil was purified by column chromatography in alumina (CH_2_Cl_2_:C_3_H_6_O 1:1), dissolved in a small amount of dichloromethane, and precipitated as the hydrochloride salt with HCl (4 M in dioxane). The solid obtained was centrifuged, washed with ethanol, and dried (**TAL3PYR·7HCl**, yield > 70%).

Anal. Calc. for C_66_H_76_N_7_Cl_7_·H_2_O: C, 64.41; H, 6.39, N, 7.97%. Found: C, 64.09; H 6.32 H%; N 7.50 N%.

^1^H NMR (300 MHz, DMSO:D_2_O 3:1): δ = 8.31–7.94 (m, 27H), 4.83 (s, 6H), 3.29 (t, *J* = 8.07 Hz, 6H), 3.16 (m, 12H), 2.79 (m, 6H), 2.24 (m, 6H).

^13^C NMR (75 MHz, DMSO:D_2_O 3:1): δ = 132.4, 131.4, 130.8, 129.9, 129.6, 129.4, 129.2, 127.9, 127.6, 126.9, 126.6, 125.7, 125.2, 124.6, 124.3, 123.3, 50.2, 48.2, 45.4, 23.0.

ESI (APCI/TOF): *m*/*z* calcd. for C_66_H_69_N_7_ [M + H]^+^: 960.5693; found 960.56.

### 2.2. Materials and Methods, Spectroscopy, DNA/RNA Interactions

The UV/vis spectra were recorded on a Varian Cary 100 Bio spectrophotometer, CD spectra on JASCO J815 spectrophotometer and fluorescence spectra on a Varian Cary Eclipse spectrophotometer at 25 °C using appropriate 1 cm path quartz cuvettes.

*Materials*. Polynucleotide was purchased as noted: calf thymus (ct)-DNA (Aldrich, St. Louis, MO, USA). Polynucleotide was dissolved in Na-cacodylate buffer, *I* = 0.05 M, pH = 7. The calf thymus (ct)-DNA was additionally sonicated and filtered through a 0.45 mm filter [36]. Polynucleotide concentration was determined spectroscopically [37] as the concentration of phosphates (equivalent to *c*(nucleobase)). Spectrophotometric titrations were performed at pH = 7.0 (*I* = 0.05 M, sodium cacodylate buffer) by adding portions of polynucleotide solution into the solution of the studied compound for fluorimetric experiments, and CD experiments were performed by adding portions of compound stock solution into the solution of polynucleotide. In fluorimetric experiments, excitation wavelengths of λ_exc_ = 334 and 351 nm were used to avoid the inner filter effect caused by the increasing absorbance of the polynucleotide. Values for log*K*_s_ were obtained by processing titration data by means of the Scatchard equation [38] (Section 3.3.3), and all have satisfactory correlation coefficients (>0.9). Thermal melting curves for DNA, RNA, and their complexes with studied compounds were determined as previously described by following the absorption change at 260 nm as a function of temperature [39]. The absorbance of the ligands was subtracted from every curve and the absorbance scale was normalized. *T*_m_ values are the midpoints of the transition curves determined from the maximum of the first derivative and checked graphically by the tangent method. The Δ*T*_m_ values were calculated subtracting the *T*_m_ of the free nucleic acid from the *T*_m_ of the complex. Every Δ*T*_m_ value here reported was the average of at least two measurements. The error in Δ*T*_m_ is ±0.5 °C.

### 2.3. Biology

#### 2.3.1. Cells

Experiments were performed using two human cell lines, namely epithelial human lung adenocarcinoma A549 (ATCC CCL-185) and human embryonic kidney HEK293 (ATCC CRL-1573). Both cell lines adhere to plastic and glass surfaces and were maintained in the cell culture under the same conditions. Cells were grown in Dulbecco Modified Eagle’s Medium (DMEM, Sigma Aldrich, St. Louis, MO, USA), supplemented with 10% of foetal bovine serum (FBS, Sigma Aldrich, USA), and incubated in the cell incubator (Thermo Fischer Scientific, Waltham, MA, USA) at 37 °C and 5% CO_2_ in a humidified atmosphere.

#### 2.3.2. Cell Viability Assay

Compound (**TAL3PYR**) was diluted in an appropriate volume of dimethyl sulfoxide solution (DMSO, Gram-Mol, Zagreb, Croatia) under sterile conditions in order to obtain a 10 mM stock solution. Solutions were kept in the dark and stored at 4 °C. For testing the cytotoxic effects of each compound on A549 cells, diluted working solutions were prepared in order to obtain a desired range of concentrations, which were tested using the MTT test [40]. Briefly, cells were seeded in 96-well tissue culture plates (7 × 10^3^ cells/well), and 24 h later they were treated with **TAL3PYR** (concentration range of 0.1–10 μM). Cells treated with the same dilutions of DMSO represented the control, while cells treated only with DMEM (10% FBS) represented the negative control. After 72 h, the media was removed, 1X MTT solution was added into each well, and the plate was incubated (37 °C, 5% CO_2_) for 3 h, allowing formazan crystals to form. The resulting MTT-formazan products were dissolved using DMSO, and their absorbance at 600 nm was measured using a microplate reader.

For determining the effect of UV exposure, cells were seeded on two plates and treated with working solutions of the respective compounds, as previously described. Plates were incubated (37 °C, 5% CO_2_) for 90 min, allowing the compounds to enter the cells. Following that, one plate was exposed to UV light (Luzchem reactor, 350 nm, 8 lamps, in total 64 W, dose 50.6 mw·m^−2^; ~18 cm lamp to cell-plate) for 5 min, 3 days in a row at the same time each day, while the other plate was left in the cell incubator in the dark and served as a control.

#### 2.3.3. Live Cell Imaging by Confocal Microscopy

Live imaging of the cells treated with compound **TAL3PYR** was performed on the A549 cell line. Cells were seeded in Ibidi imaging cell chambers (Ibidi^®^, Gräfelfing, Germany) in 500 μL of medium, 5 × 10^4^ cells/well, and wer eleft in the cell incubator for 48 h (37 °C, 5% CO_2_). Subsequently, cells were treated with a 10 μM solution of the respective compound and left in the cell incubator for 90 min to allow the compound to enter the cells. After incubation, the medium was replaced with 500 μL of fresh medium. The influence of the compounds on A549 cells before and after photoactivation (λ_exc_ = 405 nm, λ_em_ = 450–550 nm) was visualised using a Leica SP8 X confocal microscope (Leica Microsystems, Wetzlar, Germany).

#### 2.3.4. Detection of Total ROS

For detecting total ROS, A549 cells were seeded in 12-well tissue culture plates (2 × 10^5^ cells/well). After 24 h, cells were treated with a 1 μM working solution of **TAL3PYR** and incubated for 90 min (37 °C, 5% CO_2_), allowing the compound to enter to cells. The same dilution of DMSO served as a control, while H_2_O_2_ (0.002%) was used as a positive control. One plate was irradiated with UV light (350 nm) for 5 min and was analysed immediately after UV exposure. The plate that was not irradiated with UV light served as the control and was incubated in the cell incubator (37 °C, 5% CO_2_) during the whole experiment. After treatment, cells were washed with PBS, trypsinised, resuspended with cold PBS supplemented with 10% FBS (FBS-PBS), and centrifuged for 5 min (RT, 1100× *g*). Cell pellets were resuspended and washed again, but this time with cold PBS supplemented with 1% FBS (FBS-PBS), and centrifuged for 5 min (RT, 1100× *g*). After the second washing, cell pellets were resuspended in 5 µM 2′,7′–dichlorofluorescin diacetate (H2DCFDA, Sigma, USA), which is converted to dichlorofluorescin (DCF) in contact with ROS. Unstained cells were resuspended in PBS supplemented with 1% FBS. Cells were then analysed by flow cytometry using FACSCalibur (BD Biosciences, Franklin Lakes, NJ, USA).

#### 2.3.5. Plasmid Electrophoresis

For assessing the photoinduced DNA nuclease activity of **TAL3PYR**, 1 μg of pUC19 plasmid DNA (2686 pb) was incubated with 1–3 μM solutions of the compound and exposed to UV light (using a xenon arc lamp, Oriel, Stratford, CT, USA) for 60 s. Samples were run on 0.5% agarose gel and stained with Midori Green DNA binding dye (Nippon Genetics, Europe), together with samples containing the compound and plasmid DNA that were not irradiated (0 s), which served as a control for assessing photoinduced nuclease activity of the compound. Plasmid without the compound was irradiated with UV light under the same conditions to determine whether UV light itself induces changes in plasmid DNA forms (linear and supercoiled form). After gel electrophoresis, plasmid DNA forms were visualised on a UVITEC Imager (Cleaver Scientific, Rugby, UK).

#### 2.3.6. Cell Cycle Analysis

A549 cells were seeded in 12-well tissue culture plates (2 × 10^5^ cells/well). After 24 h, cells were treated with 0.8 and 0.1 μM working solution of **TAL3PYR** and incubated for 90 min (37 °C, 5% CO_2_), allowing compounds to enter the cells. The concentration of 0.8 μM represents IC50 of irradiated **TAL3PYR**, according to MTT assay. The working solution of 100 μM H_2_O_2_ served as a positive control, while cells treated with the same dilution of DMSO represented negative control sample. Cells were irradiated once for 15 min as described in the cytotoxicity assay, while non-irradiated cells served as a control and were incubated in the cell incubator (37 °C, 5% CO_2_) during the whole experiment. After irradiation, cells were incubated for the next 24 h (37 °C, 5% CO_2_) and then trypsinized, centrifuged (5 min, RT, 1200 rpm), and washed twice with PBS. After the last centrifugation, the supernatant was discarded, and cell pellets were put on ice. Cells were fixated with cold ethanol (96%), which was gently added to the cell pellets. During the fixation step, samples were gently vortexed in order to distribute ethanol equally through the whole pellet. After fixation, samples were incubated at −20 °C overnight. The next day samples were centrifuged (5 min, 4 °C, 1500 rpm) and the supernatant was discarded. The cell pellet was washed with PBS and centrifuged (5 min, 4 °C, 1200 rpm) twice. After the second wash, 10 μg/μL of RNAse A solution was added to cell pellets and incubated at 37 °C for 30 min. Next, 50 μg/mL of propidium iodide (PI) was added to each sample at the same ratio as RNAse A. Samples were stored on ice in the dark and analysed immediately using FACSCalibur (BD Biosciences, USA). Data were analysed using Flow Logic software (8.4).

#### 2.3.7. Antiviral Activity of TAL3PYR

End Point Dilution Assay was used to determine the antiviral activity of **TAL3PYR** against Human Adenovirus Type 5 (HAdV5). HEK-293 cells were seeded in a 96-well tissue culture plates (1 × 10^4^ cells/well) in DMEM (10% FBS) and left overnight in the incubator (37 °C, 5% CO_2_). After 24 h, HAdV5 (2.9 × 10^9^ viral particles) was incubated in DMEM (10% FBS) solution of the compound **TAL3PYR** (1 µM) or DMSO in the same concentration. Solutions were incubated at 37 °C for 15 min, followed by UV irradiation (15 min), while non-irradiated virus served as a control. Following that, 3-fold serial dilutions in DMEM (10% FBS) were prepared and added in octuplicates to HEK-293 with a starting dilution of 1 × 10^−8^. After 10 days of incubation, all wells showing a cytopathic effect (CPE) were counted using an inverted light microscope (Optika, Via Rigla, Italy), and virus titre was calculated [41].

#### 2.3.8. Statistical Analysis

Each experiment was repeated at least three times unless otherwise noted. The data were analysed by the unpaired Student’s *t*-test and expressed as means ± standard deviation (SD). Data were considered statistically significant at a *p*-value < 0.05.

## 3. Results

### 3.1. Synthesis

**Scheme 2:**
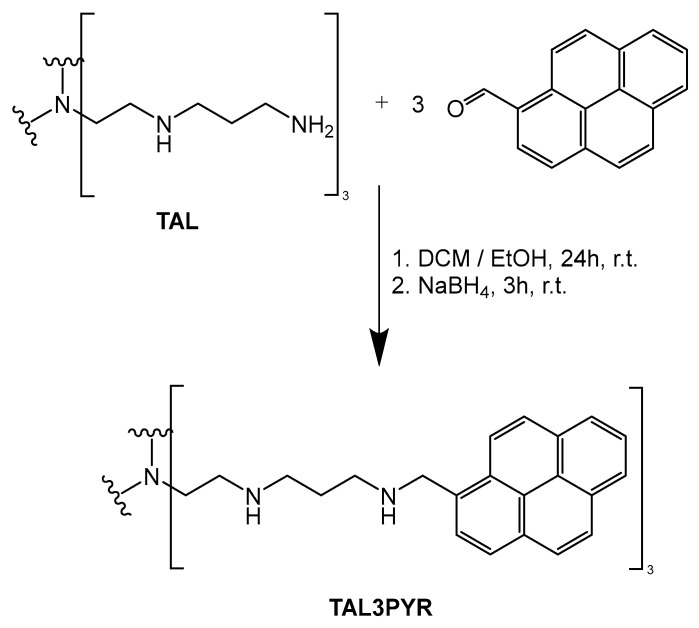
Synthesis pathway for the preparation of **TAL3PYR**.

The studied compound **TAL3PYR** was prepared by a synthetic strategy similar to the strategy previously described for condensed aromatics analogues (Scheme 2, **ATAL**, **N3TAL**) [20].

In order to obtain the fully functionalized tripodal ligand, we reacted TAL in its free amine form with just slightly over three equivalents (1% excess) of the 1-pyrenecarboxaldehyde. The obtained product was reduced in situ with NaBH_4_ and was processed to obtain the final product in powdered form as the hydrochloride salt.

### 3.2. Physico-Chemical Properties of TAL3PYR in Aqueous Solutions at pH = 7.0

**TAL3PYR** is soluble in pure water (*c* = 5.0 × 10^−3^ M), allowing further experiments to be performed without addition of other solvents. The pH dependence of **TAL3PYR** expected to be similar to previously studied tripodal ligands, particularly to the naphthalene analogue **N3TAL** (Scheme 2) [20]. Thus, for further studies the biologically relevant buffered solution was chosen (Na-cacodylate buffer at pH = 7, *I* = 0.05 M), at which **TAL3PYR** should have five positive charges. As a reference compound we used 1-pyrenebutyric acid (**PYR-Bz**), representing pyrene substituted at 1 position with aliphatic residue. The **PYR-Bz** stock solution (0.01 M) was prepared in DMSO and for the experiments it was further diluted in the buffer solution, taking care that the volume ratio of DMSO was less than 1%.

The studied compound solution in the buffer was stable for a week, after which a minor decrease in UV/Vis spectrum was noticed. For that reason, at the beginning of the week, new solutions were prepared. The UV/vis spectrum of **TAL3PYR** was proportional to concentrations up to *c* = 2.0 × 10^−5^ M (Supplementary Information, Figure S1). Absorption maxima and corresponding molar extinction coefficients (ε) are given in Table 1.

**Figure 1:**
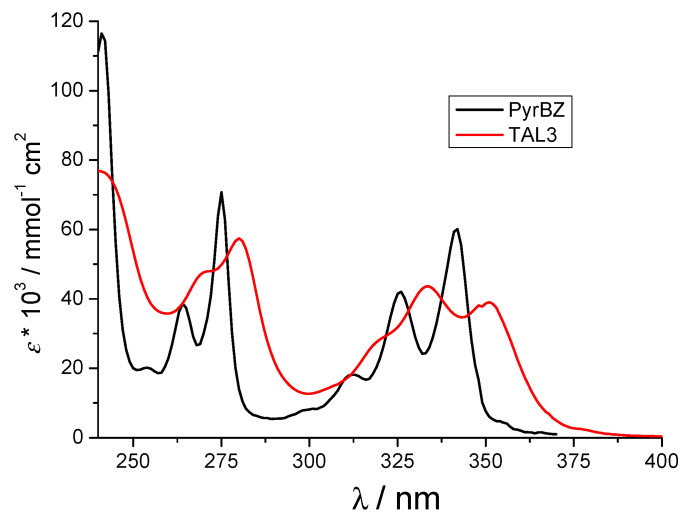
Comparison of UV/Vis spectra of **TAL3PYR** (*c* = 2.0 × 10^−5^ M) and reference compound **PyrBZ** (*c* = 2.0 × 10^−5^ M) at pH = 7.0, sodium cacodylate buffer at 25 °C.

Changes of the UV/Vis spectra with the temperature were negligible up to 55 °C, however, a rise in temperature above 55 °C resulted in an increase in the number of aggregated particles (opalescent solution, Supplementary Information, Figure S2). The reproducibility of UV/Vis spectra upon cooling back to 25 °C was good, except at 236 and 241 nm absorption maxima wavelengths, where a decrease of absorption was noticed (<9%, Supplementary Information, Figure S2). Comparison of the UV/Vis spectra of **TAL3PYR** and of the reference pyrene benzoic acid (**PyrBZ**) revealed that absorption maxima of **TAL3PYR** (Figure 1) are strongly bathochromically shifted and broadened with a hypochromic effect. Additionally, losing the sharp vibronic structure of **PyrBZ** peaks is typical for a free pyrene absorption. All these effects suggest that the pyrenes of **TAL3PYR** are strongly aromatically stacked.

**Figure 2:**
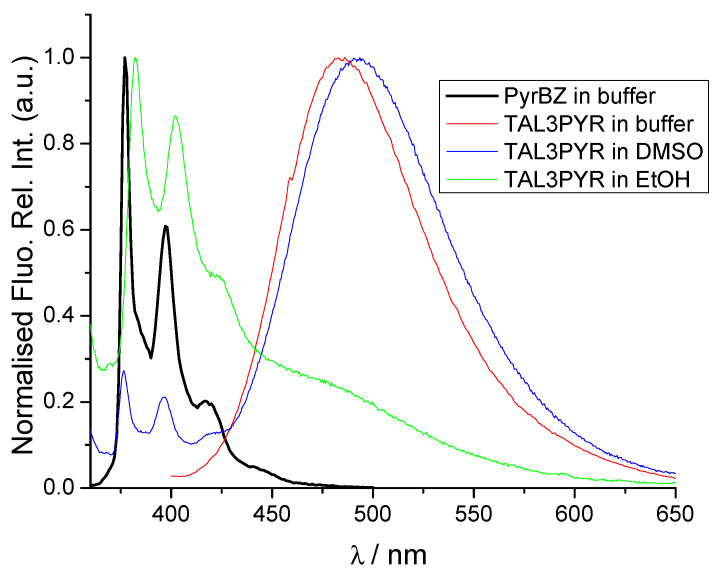
Normalized fluorescence emission spectra of referent **PyrBZ** (▬, λ_exc_ = 342 nm, 5.0 × 10^−7^ M, at pH = 7.0, sodium cacodylate buffer, *I* = 0.05 M) and **TAL3PYR** (λ_exc_ = 351 nm, 5.0 × 10^−7^ M) in buffer (▬, pH = 7.0, sodium cacodylate buffer, *I* = 0.05 M), DMSO (▬), and EtOH (▬).

The emission spectrum of **TAL3PYR** in buffered aqueous solution (sodium cacodylate buffer, *I* = 0.05 M, pH = 7.0) showed a dominant maximum at about 500 nm, characteristic for pyrene excimer [21], at variance to reference compound **PyrBZ**, which only shows the emission of the free pyrene (Figure 2). The strong stacking of pyrenes in **TAL3PYR** was further corroborated by a strong concentration and temperature dependence of the emission. For instance, an increase of the **TAL3PYR** concentration from 2.5 to 5.0 × 10^−7^ M resulted in an emission blue shift (from 492 nm → 486 nm, Figure S3, Supplementary Information), whereas heating of the latter solution yielded an opposite shift of emission maximum (Supplementary Information, Figure S3).

Intriguingly, the emission of **TAL3PYR** dissolved in EtOH (Figure 2) resembles the emission of **PyrBZ**, thus consisting at least partially of the emission of the monomer pyrene fluorophore not forming an excimer [21]. However, **TAL3PYR** dissolved in DMSO showed similar emission of pyrene excimer at 500 nm, as was found for aqueous solution, but with a minor presence of free pyrene emission at 380–420 nm range (Figure 2). Such solvatochromic effects on **TAL3PYR** emission prompted us to study the photophysical properties in more detail using time-correlated single photon counting (TC-SPC, Supplementary Information, Figure S7), determining the absolute quantum yields of fluorescence (*Φ_f_*, Table 1).

The much higher quantum yield value obtained for **TAL3PYR** in EtOH solution was compared with the aqueous solution, suggesting a strong stacking of the pyrene moieties in water, which indicates a more efficient non-radiative decay of excited molecules.

Analysis of the fluorescence emission decay times obtained for **TAL3PYR** in various solvents and at various emission wavelengths revealed a significant difference in comparison to the pyrene-benzoic acid **PyrBZ** (Table 1) used as a reference. A dominant decay time of **PyrBZ** (τ = 100.3 ns; 99.2%) agrees well with the pyrene-fatty acid analogues, typically displaying decay times near 100 ns [21]. However, decay times of **TAL3PYR** are significantly shorter and more complex, mostly characterised by negative pre-exponential factor (−B), which is a strong indication for the formation of aggregates in the excited state [42,43]. A single pyrene monomer should form excimer controlled by diffusion [42]. However, this ideal situation is very rarely observed because the crowded geometry of the **TAL3PYR** containing three pyrenes introduces constraints and heterogeneities, which affects the process of excimer formation. As a result, several different combinations of excimer could be formed.

Accordingly, in the non-aqueous solvents (DMSO, EtOH), decay times collected at 486 nm (maximum wavelength of the excimer emission) show two decay times, one similar for both solvents (τ = 41–45 ns), and another one either shorter (τ = 4.22 ns, EtOH) or much longer (τ = 59.19 ns, DMSO), but none of them reach the value for the pyrene monomer **PyrBZ** (τ = 100.3 ns). An even stronger solvent dependence is evident for **TAL3PYR** aqueous solution, with a very short decay time of τ = 3.84 ns agreeing well with short decay times of EtOH, while the longer decay time of τ = 21.00 ns agrees with value τ = 22.29 ns, collected for EtOH at 400 nm maximum (considered dominant for pyrene non-aggregated species). Thus, in all solvents, **TAL3PYR** pyrenes are at least partially in excimeric form (mostly two different excimers), and empirically we can attribute short decay times of **TAL3PYR** (τ = 4–5 ns) to aqueous or ethanolic solutions, whereas the longest one (τ = 59.19 ns) is specific for the DMSO.

In addition, we also observed time-dependent changes in emission of **TAL3PYR** aqueous solution, triggered only by mixing the solution (no changes if solution was kept still), whereby readings of fluorescence intensity randomly varied for up to 10% after every mixing. In parallel, collected UV/Vis spectra did not change, excluding the chemical decomposition of molecules or eventual precipitation. No clear time-dependent trend in emission change was observed, thus we attributed these fluctuations to intra- and/or intermolecular processes of aggregation/de-aggregation of pyrenes of **TAL3PYR** induced by light irradiation and mechanical stirring. Such a combined input could initiate formation of various pyrene-excimer species, as evidenced by the discussion above of at least two different decay lifetimes, and negative pre-exponential factors (−B). The complex system, including at least three pyrenes in **TAL3PYR** or even more in the case of intermolecular interactions, could not be simply deconvoluted, but would eventually require a laborious set of experiments and analysis [42,43], which was out of the scope of this biologically-oriented work.

### 3.3. Study of Interactions of TAL3PYR with ds-DNA and ds-RNA

#### 3.3.1. Thermal Denaturation Experiments

Ds-polynucleotide buffered solutions dissociate in two single stranded sequences upon heating, usually at a well-defined temperature (*T*_m_ value), commonly used for the characterisation of various ds-DNA or ds-RNA related processes [39]. For instance, the binding of small molecules to ds-polynucleotides usually increases the thermal stability of the ds-helices, resulting in positive ∆*T*_m_ value, which can be correlated with the various binding modes. However, binding mode has to be confirmed by an independent method.

For most pyrene analogues, classical intercalation into ds-DNA should cause stabilisation with Δ*T*_m_ > 5 °C due to aromatic stacking interactions with DNA base pairs, whereas the binding of pyrenes within the polynucleotide groove, usually driven by hydrophobic effects, commonly has a negligible stabilising outcome [44].

**Figure 3:**
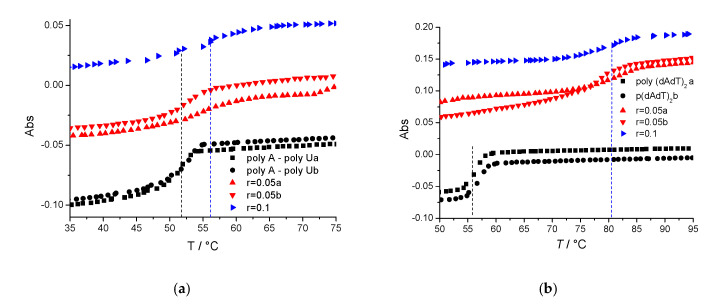
Thermal denaturation curves of (**a**) poly A—poly U (*c*(AU-RNA) = 2.5 × 10^−5^ M, r_[**TAL3PYR)**]/[AU-RNA]_ = 0.05; 0.1); (**b**) AT-DNA (*c*(AT-DNA) = 2.5 × 10^−5^ M, AT-DNA (r_[**TAL3PYR)**]/[DNA]_ = 0.05; 0.1). Performed at pH 7.0, sodium cacodylate buffer, *I* = 0.05 M.

The addition of **TAL3PYR** to ds-DNA or ds-RNA resulted in the strong stabilisation of double-stranded polynucleotide, even at low **TAL3PYR**/polynucleotide ratio r ≤ 0.1, (Figure 3, Table 2), while at higher ratios (r > 0.1) precipitation occurred, most likely as a consequence of DNA/RNA negative charge neutralisation by the strongly positively charged **TAL3PYR** (Table 1).

Detailed analysis of stabilisation effects revealed strong selectivity toward AT-DNA with respect to AU-RNA. Since these two polynucleotides differ mainly by secondary structure of double helix (AT-DNA being typical B-helix, while AU-RNA is A-helix, see Table S1 in Supplementary Information), such selectivity can be attributed to the difference in binding site of **TAL3PYR**. Namely, the AT-DNA minor groove is ideally suited for the binding of small molecules, at variance to the very wide and shallow minor groove of AU-RNA. On the other hand, the major groove of RNA is of a similar width to the DNA minor groove, but is much deeper, and the binding of **TAL3PYR** into RNA major groove would allow poor contact with negative RNA backbone.

Further, the difference in stabilisation of both DNA and RNA at ratios r = 0.05 and 0.1 is very small, suggesting that all dominant binding sites along the DNA/RNA helix are occupied by **TAL3PYR** between these two ratios.

#### 3.3.2. Spectrophotometric Titrations of Pyrene Analogues with DNA, RNA

The addition of ct-DNA to **TAL3PYR** buffer solution resulted in the increase of the UV/Vis spectrum, accompanied by an increase of the baseline at wavelengths above 400 nm (Figure S5 in the Supplementary Information), suggesting colloidisation of the solution, likely caused by aggregation of **TAL3PYR**/DNA complex into large aggregates. This can be attributed to highly positively charged **TAL3PYR** molecules, neutralising negatively charged DNA backbone, resulting in a neutral, highly hydrophobic complex. Such an event hampered the accurate processing of the titration data and calculation of a binding constant; however, an affinity in the order of logK ~6 could be estimated.

**Figure 4:**
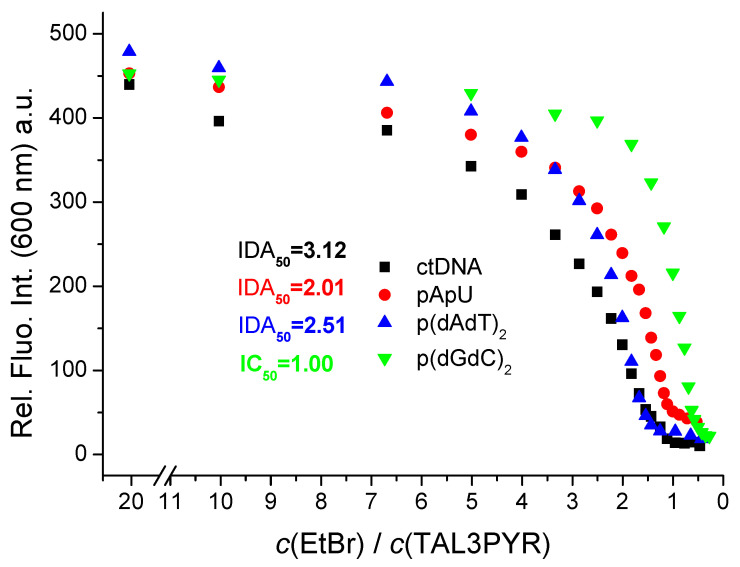
Changes in fluorescence of ethidium bromide EtBr (*c* = 5 × 10^−6^ M, λ_exc_ = 505 nm, λ_em_ = 600 nm) from the complex with the various ds-DNA or ds-RNA (*c* = 5 × 10^−5^ M): upon addition of **TAL3PYR**. The IDA_50%_ value is calculated as the ratio [EtBr]/[**TAL3PYR**] = (Int(EtBr − DNA complex) − Int(EtBr))/2. Performed at pH 7.0; sodium cacodylate buffer, *I* = 0.05 M.

Unstable emission of **TAL3PYR** aqueous solution upon mixing the sample (see discussion below Table 1) prevented accurate fluorimetric titrations with various ds-DNA or ds-RNA, so we opted for Indicator Displacement Assay (IDA) methodology [45] using ethidium bromide (EtBr) displacement assay. A highly emissive EtBr/polynucleotide complex was prepared and subsequent systematic addition of **TAL3PYR** caused EtBr displacement, as evidenced by the decrease of the EtBr maximum emission (Figure 4) [46,47]. The **TAL3PYR** efficiently displaced EtBr from ds-DNA/RNA and calculated IDA50% (Indicator Displacement Assay) values (Figure 4) corroborated the results of thermal denaturation experiments, supporting a very strong binding of **TAL3PYR** to ds-DNA/RNA.

According to the previously determined binding constant for EtBr in identical conditions (log*K_s_* = 6 [37]), IDA_50%_ values determined for **TAL3PYR** suggest approximately two-fold higher affinity for most of the DNA/RNAs in comparison to EtBr. However, since EtBr is a DNA/RNA intercalator and **TAL3PYR** most likely binds within DNA/RNA grooves, IDA_50%_ values give only an indirect estimate of the **TAL3PYR** affinity.

The comparison of the IDA_50%_ values and thermal denaturation results (Table 2) for AU-RNA revealed that **TAL3PYR** very efficiently displaced EtBr (IDA_50%_ = 2) but weakly stabilised RNA against thermal denaturation. Such effects would suggest that **TAL3PYR** binds within the very deep RNA major groove due to mostly hydrophobic interactions, with only marginal electrostatic interaction with RNA backbone. This was at variance with the **TAL3PYR** binding into minor groove of DNA (Supplementary Information Table S1), which is much shallower and allows for strong electrostatic interaction with the DNA backbone.

The somewhat lower IDA_50%_ value observed for GC-DNA can be attributed to the sterically hindered minor groove (by protruding amino groups of guanine (Supplementary Information Table S1), which does not allow for the deep insertion of **TAL3PYR**.

#### 3.3.3. Circular Dichroism (CD) Experiments

Circular dichroism (CD) spectropolarimetry as chirooptic spectrophotometry is highly sensitive to conformational changes in chiral ds-DNA or ds-RNA helices, and us thus commonly used to monitor the changes in the secondary structure of polynucleotides. Furthermore, achiral small molecules, such as **TAL3PYR**, do not have intrinsic CD spectrum, but can acquire an induced CD spectrum (ICD) upon binding in one dominant mode to polynucleotides, which can provide useful information about the modes of interaction [48,49].

**Figure 5:**
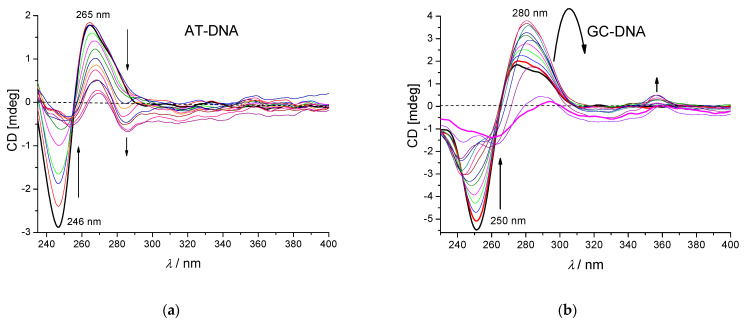

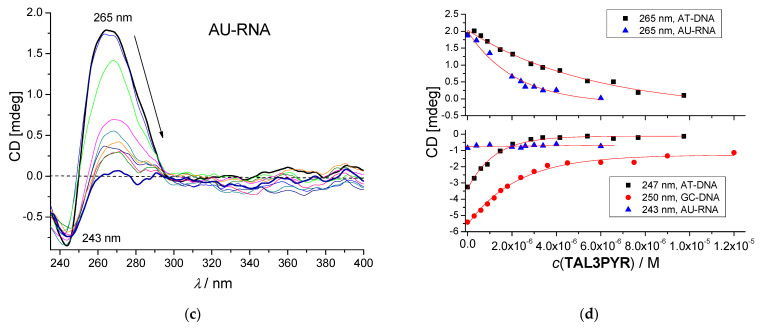
CD titration of various ds-DNA/RNA (*c* = 3.0 × 10^−5^ M): (**a**) AT-DNA, (**b**) GC-DNA, (**c**) AU-RNA, with **TAL3PYR** (different colour lines in **a**–**c**) given for better visibility of titration course); (**d**) dependence of CD response at maxima on *c*(**TAL3PYR**), performed at pH = 7.0, buffer sodium cacodylate, *I* = 0.05 M.

The addition of **TAL3PYR** induced strong changes in CD spectra of all studied ds-DNA or ds-RNA (Figure 5a–c, Supplementary Information Figure S6). Such a strong decrease of DNA/RNA CD spectra suggests almost a complete loss of double helix chirality, likely caused by the neutralisation of DNA/RNA negative charges by positively charged **TAL3PYR** and the consequent collapse of helical secondary structure. Here we did not observe appearance of the ψ-DNA, as we reported for the close analogue **L2** (Scheme 1, [18]).

Pronounced changes of CD bands in 240–290 nm range allowed for processing the titration data by means of the Scatchard equation (Figure 5d) to calculate binding constants (Table 3). For AT-DNA, isoelliptic points were observed within the ratio r_[**TALPYR**]/[DNA]_ = 0.01–0.2, supporting the formation of only one dominant type of a complex (Figure 5a), which allowed for the calculation of a binding constant [38]. Accordingly, binding constants were calculated for the titration of other polynucleotides with **TAL3PYR** (Figure 5d).

The binding constants calculated from CD titrations agreed excellently with the values obtained from IDA_50_ experiments (Table 3, Figure 4). A comparison with previously studied polyamine analogue **L1** (Table 3, Scheme 1) revealed that although **TAL3PYR** and **L1** have similar net positive charge (from +5 to +6), the intramolecular aggregation of pyrene units in TAL3PYR prevented a fully efficient electrostatic interaction with the negatively charged DNA backbone, resulting in a decreased affinity toward ds-DNA/RNA of an order of magnitude. However, the **BFTA**-analogue, characterised by two large aromatic units and only 3+ positive charge, showed only one order of magnitude lower affinity with respect to **TAL3PYR**, demonstrating that aromatic interactions with DNA can compensate its lower positive charge.

Within the 300–350 nm range in the CD spectra, at which only pyrene absorbs, weak positive-induced (I) CD bands were observed for AT- and GC-DNA. However, since pyrenes are mostly intramolecularly aggregated, it was impossible to relate the sign of the ICD band with the binding site [48,49].

The analysis of all data, including spectrophotometric titrations, thermal denaturation experiments, as well as structural features of bulky intramolecularly aggregated **TAL3PYR**, supports binding into DNA/RNA grooves (Table S1, Supplementary Information) as the dominant binding mode.

#### 3.3.4. TAL3PYR Induces Plasmid DNA Cleavage

**Figure 6:**
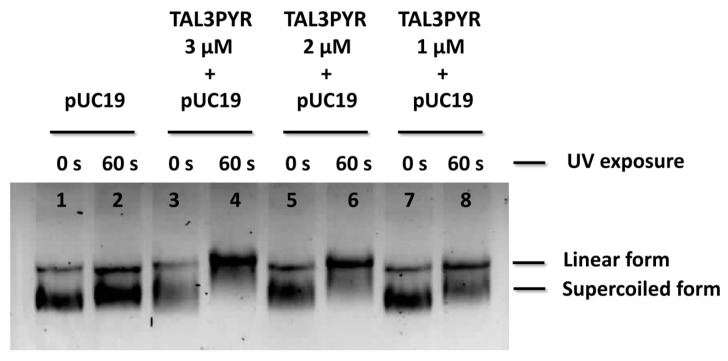
A representative agarose gel showing photo-cleavage as a function of time for 38 µM supercoiled bp pUC19 DNA in 10 mM sodium cacodylate buffer pH 7.0 (22 °C). Individual reactions in the presence and absence of **TAL3PYR** (*c* = 1, 2, 3 µM) were irradiated with a 300–350 nm UV lamp for 60 s.

Due to its photophysical properties, allowing photo-induced generation of singlet oxygen [21,22] as well as strong binding to ds-DNA (vide supra), the pyrene moieties of **TAL3PYR** can cause DNA cleavage upon UV irradiation [28,29]. In order to determine whether **TAL3PYR** possesses DNA photo cleavage properties, we assessed 38 µM (bp) of pUC19 plasmid DNA cleavage in the presence of **TAL3PYR** with or without UV irradiation (Figure 6).

The rationale behind this choice is based on the circular structure of native plasmid DNA (most common form of plasmid DNA). When DNA cleavage occurs, this circular molecule linearizes. This transition from a coiled DNA to its linear form presents a unique advantage in visual analysis when employing DNA electrophoresis. Namely, the intact, circular plasmid DNA, due to its compact structure, migrates more rapidly through the agarose gel upon the application of an electric current in comparison to linearized DNA, being more extended, giving greater resistance and migrating at a slower rate. As a result, the distinction between intact and cleaved DNA becomes remarkably evident, as demonstrated by the clear difference in band positions on the gel [50].

In Figure 6, the dose-dependent differences in band appearance are indicative of the extent of DNA cleavage. As the concentration of the compound increases, the intensity and pattern of the bands change, which corresponds to a higher rate of DNA cleavage upon UV exposure. At a molecular level, **TAL3PYR** obviously interacts with the DNA structure and, when subjected to UV radiation, triggers a breakage in the DNA strands.

## 4. Biological Activity of TAL3PYR

### 4.1. UV Light Irradiation Increases the Cell Toxicity of TAL3PYR

Prior experiments in this work showed that **TAL3PYR** strongly binds to ds-DNA, which, if happens in the cells, can be a cause of strong cytotoxicity [35]. Also, the photoinduced-DNA cleavage results (Figure 6) indicate that if **TAL3PYR** enters the cells, it can cause severe damage close to the intracellular locus under photo irradiation [21,22,23].

**Figure 7:**
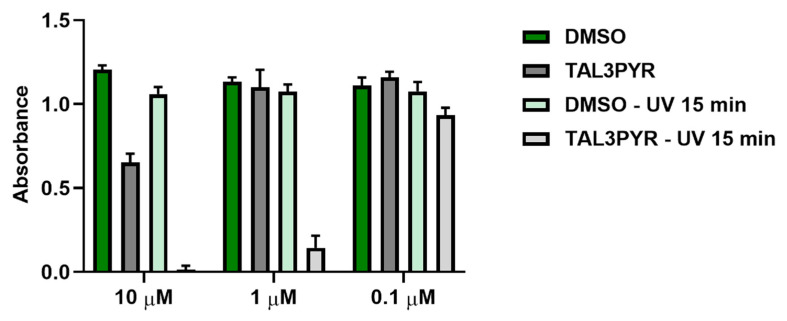
Cell survival of A549 cells treated with different concentrations of **TAL3PYR**, with or without UV light irradiation. Data are presented as mean ± SD made in four replicates, relative to the control samples. Control samples consisted of cells treated with DMSO at the same concentration as a tested compound. Representative data of three independent experiments which yielded similar results are shown.

Thus, we studied the impact of the addition of **TAL3PYR** to human lung carcinoma (A549) cell line by MTT assay (Figure 7), compared to the control cells (non-treated by compound).

Treating cells with **TAL3PYR** only showed minor toxicity at the highest tested concentration (10 μM). However, when **TAL3PYR**-treated cells were exposed to the UV light (Luzchem reactor, 350 nm, 8 lamps, in total 64 W, Dose 50.6 mw·m^−2^; ~18 cm lamp to cell-plate) for 15 min, the toxicity dramatically increased. Under the same conditions, the irradiation of non-treated cells did not induce any measurable effect on their viability.

These results indicate that **TAL3PYR** binds to the cells, and the particularly strong effect of very short exposure to light (15 min) supports the efficient uptake of **TAL3PYR** inside cells. However, additional experiments are needed for the better characterisation of biological effects.

### 4.2. UV Irradiated TAL3PYR Induces Change in Cell Morphology, Induces ROS Production, and Causes Changes in the Cell Life Cycle

Next, we performed **TAL3PYR** irradiation experiments on the confocal microscope by using maximum power laser irradiation at λ_exc_ = 405 nm, monitoring the morphological changes of irradiated cells in the bright-field mode. **TAL3PYR** efficiently entered treated cells within 90 min of incubation (evidenced by pale blue fluorescence in the cytoplasm); however, due to the weak emission and interference of cellular autofluorescence backgrounds, no accurate collocalisation staining could be performed. When the intensity of the incident light was increased from 10% to 100% (0.5 mW), after 1 min, cells treated with **TAL3PYR** showed severe blebbing and disintegration. These intensive morphological changes resemble ROS species production and consequent cellular damage. Intriguingly, cells placed outside the light-source focus circle were not affected, thus photo-induced damage experiments could be repeated many times by simply shifting laser focus over the same sample of living cells treated with **TAL3PYR**. In the control experiment (no **TAL3PYR** added), irradiation did not yield any observable effect. This allowed us to hypothesise that the effect observed in **TAL3PYR**-treated cells exposed to UV light can be attributed to the well-known ability of pyrene to produce singlet oxygen in biological medium, stimulating a cascade reaction and yielding various reactive oxygen species (ROS).

Therefore, the generation of ROS in A549 cells treated with 1 μM of **TAL3PYR**, with or without exposure to UV light, was assessed. The method relies on the ability of the cell-permeant 2′,7′-dichlorodihydrofluorescein diacetate (H2DCFDA) that upon cleavage of the acetate groups by intracellular esterases and oxidation, the nonfluorescent H2DCFDA is converted to the highly fluorescent 2′,7′-dichlorofluorescein (DCF). Therefore, ROS species detected by DCF fluorescence are most likely localized within the cell.

**Figure 8:**
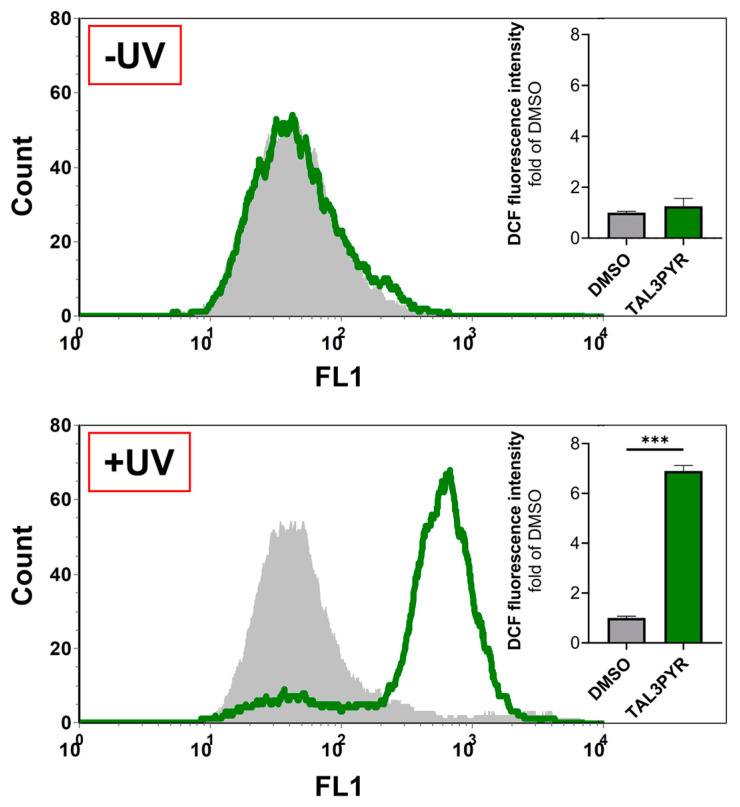
The amount of ROS in A549 cells treated with 1 μM **TAL3PYR**, with or without exposure to UV light. Levels of ROS were assessed by measuring the fluorescence intensity of DCF by flow cytometry. A representative data of two independent experiments which yielded similar results is shown. *** *p* < 0.001 (Student’s *t* test).

Figure 8 shows that **TAL3PYR** itself did not induce ROS production, as evidenced by the absence of any change in the fluorescence intensity of dichlorofluorescin (DCF) after **TAL3PYR** treatment without UV irradiation. However, after UV irradiation of **TAL3PYR**-treated cells, the fluorescence intensity of DCF was 7-fold higher compared to DMSO-treated cells, showing the strong production of ROS induced by UV irradiation.

Additionally, ROS, especially species such as hydroxyl radicals and singlet oxygen, have extremely short half-lives and limited diffusion distances [51]. This means they react very close to their site of generation. If ROS were generated outside the cell, they would have to pass through the cell membrane before reacting with DCFH. Given their highly reactive nature, it is more probable that they would react with other molecules before internalizing inside the cell. This reinforces the idea that ROS detected by DCF fluorescence are primarily those produced intracellularly.

**Figure 9:**
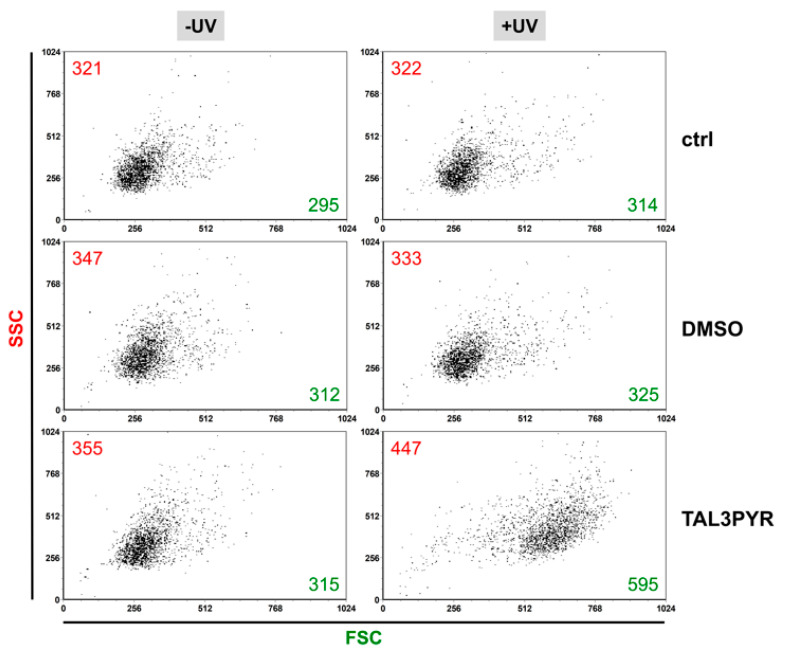
Cell morphology of A549 cells treated with 1 μM **TAL3PYR**, with (+UV) or without (−UV) irradiation determined by flow cytometry. Average forward scatter (FSC) intensity is shown in green and the average side scatter (SSC) intensity is shown in red for each condition used. Control condition represents non-treated cells. Representative data of two independent experiments, which yielded similar results, are shown.

Since increased ROS production can be accompanied by cell damage, we also studied changes in cell morphology of **TAL3PYR** treated cells. While UV light irradiation of DMSO-treated cells did not cause significant changes in cell morphology, UV light irradiation of **TAL3PYR**-treated cells demonstrated increased side scatter (SSC) intensity and forward scatter (FSC) intensity, meaning that cells were larger in diameter and have a greater internal complexity, i.e., granularity (Figure 9). These data confirm previously observed blebbing by confocal microscopy.

**Figure 10:**
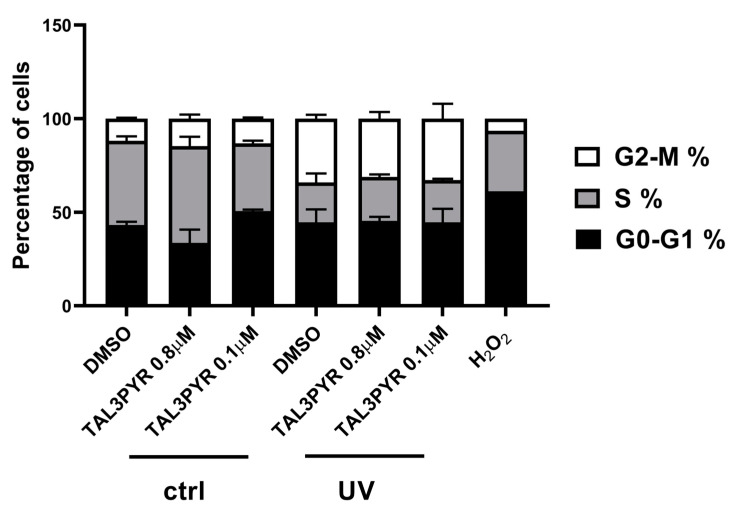
Effect of **TAL3PYR** on the cell cycle. **TAL3PYR** treated cells, with or without UV light exposure, were harvested, and the cell cycle was analysed by flow cytometry. Cells treated with DMSO served as negative control, while cells treated with H_2_O_2_ served as positive control. Results are presented as percentage of cells ± SD made in duplicates. The representative data of two independent experiments which yielded similar results are shown.

Next, we examined the cell cycle of A549 cells treated with different concentrations of **TAL3PYR** followed by UV light exposure. As shown in Figure 10, **TAL3PYR** treatment had no significant influence on cell cycle regardless of UV light exposure. We did observe that the treatment of A549 with 0.8 μM of **TAL3PYR** (IC_50_ for irradiated **TAL3PYR**) without UV exposure slightly induced an increase in the cell population arrested in S phase compared to control cells treated with DMSO (51.74% vs. 45.01%, respectively), however this change is within standard deviations. An arrest in G2-M phase which can be seen in UV light-irradiated **TAL3PYR** treated cells is comparable to UV light-irradiated cells treated only with DMSO, i.e., control cells, leading us to conclude that UV light-irradiated **TAL3PYR** has no effect on cell cycle. Of note, many studies using cultured cells have reported both G0-G1 and G2-M phase cell cycle arrests as responses to DNA damage induced by UV radiation itself [52,53]. As expected, treating A549 with 100 μM of H_2_O_2_ induced cells cycle arrest in the G1 phase compared to control cells treated with DMSO alone (61.17% vs. 43.14%, respectively). Treatment with H_2_O_2_ represented positive control for cycle arrest analysis [54].

### 4.3. TAL3PYR Forms a Complex with HAdV5 but Shows No Antiviral Activity

**Figure 11:**
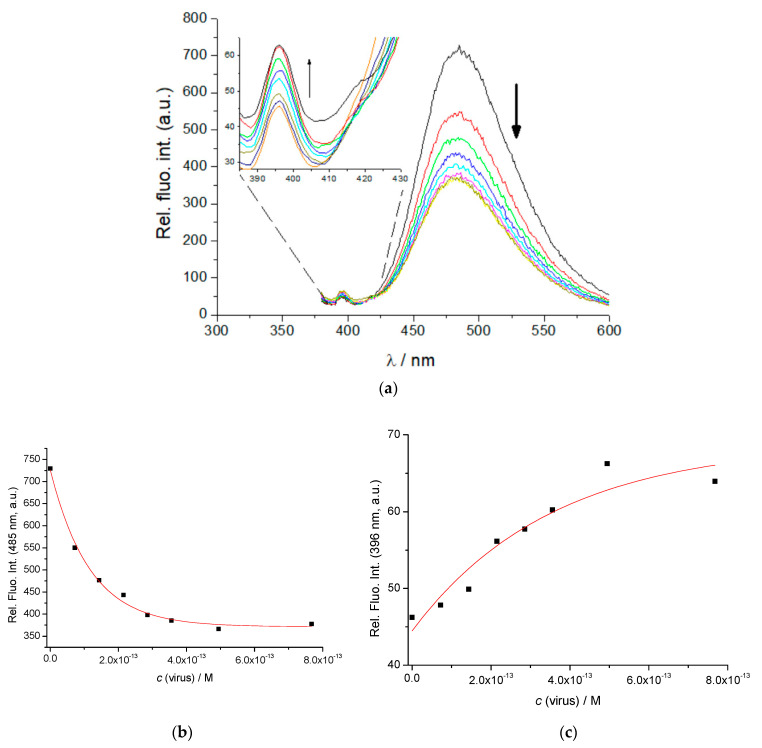
(**a**) Changes in fluorescence spectra of **TAL3PYR** (*c* = 5.0 × 10^−7^ M, λ_exc_ = 334 nm; incubation time 30 s.) upon titration with *HAdV5* virus (different colour lines in (**a**) given for better visibility of titration course). Dependence of **TAL3PYR** emission at (**b**) λ_max_ = 486 nm and (**c**) 396 nm; on *c*(*HAdV5*), at pH 7.0, sodium cacodylate buffer, *I* = 0.05 M.

We also tested the action of **TAL3PYR** against Human Adenovirus Type 5 (*HAdV5*), commonly used as gene delivery vector. Intriguingly, preliminary test of adding aliquots of virus stock solution to **TAL3PYR** in buffered medium resulted in a strong change of **TAL3PYR** emission, which remained stable even upon prolonged mixing and exposure of excitation light, contrary to the unstable emission of the free **TAL3PYR** (vide supra, Section 3.2) or its complex with DNA/RNA. Such stable emission of **TAL3PYR**/*HAdV5* complex allowed us to perform the fluorimetric titration of **TAL3PYR** by virus stock solution, monitoring the strong decrease of emission at 485 nm (characteristic for aggregated pyrene form) but also simultaneously in the two-fold increase of emission at wavelength characteristic for the single pyrene emission: λ = 396 nm (Figure 11a; enlarged part).

Excellent fitting of the titration data at both 485 nm and 396 nm (Figure 11b,c), respectively), to the first exponential equation strongly supported the formation of non-covalent **TAL3PYR**/*HAdV5* complex, suggesting that for *c*(**TAL3PYR**) = 5 × 10^−7^ M at 100-fold dilution of virus stock solution, almost 100% of the **TAL3PYR**/*HAdV5* complex was formed, and even at 1000-fold dilution about 50% of complex is present. Since we could not determine binding stoichiometry of **TAL3PYR**/*HAdV5* complex, the apparent binding constant (presuming 1:1 stoichiometry) of *K_app_* ~10^7^ M^−1^ could be estimated.

Further, heating of the **TAL3PYR**/*HAdV5* complex from Figure 11 experiment to 90 °C and cooling back to room temperature almost fully recovered the emission spectrum of 100% complexed **TAL3PYR**/*HAdV5* complex. Thus, the fluorescence of **TAL3PYR**, when bound to virus, does not depend on virus denaturation–renaturation, which suggests that binding site is well-preserved.

**Figure 12:**
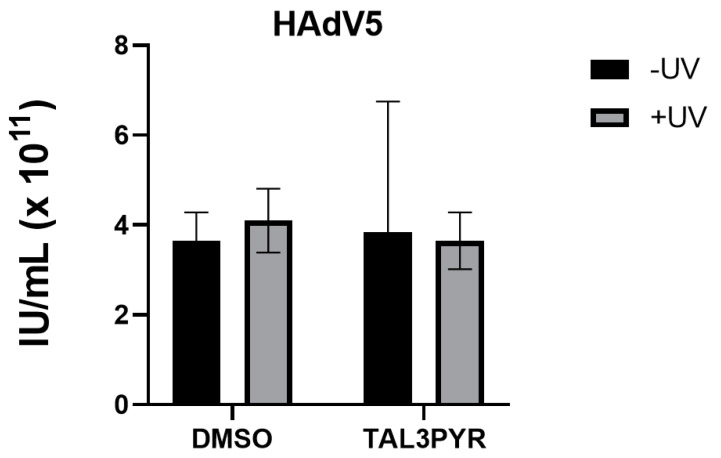
The effect of the addition of **TAL3PYR** on *HAdV5* infectivity, in the dark, and under UV irradiation. Results are presented as mean ± SD of two biological replicas with eight technical replicates in each condition.

Following that, we tested the impact of **TAL3PYR** addition on virus infectivity. **TAL3PYR** was added to the viral stock solution in quantity to strongly bind to viral particles (relying on the results on Figure 11), the solution was split into two separate samples, and one part was irradiated by UV light (300–350 nm, 15 min). Both viral stock solutions were added to the cells and virus infectivity was assayed. Results in Figure 12 showed that the addition of **TAL3PYR** did not influence the number of infective viral particles (IU/mL). It is possible that the binding site of **TAL3PYR** is in the hydrophobic regions of the virus proteins, while most regions of the virus proteins, such as the knob domain of the fibre protein, important for the initial interactions with cell receptors, are highly hydrophilic [55]. Therefore, although **TAL3PYR** makes a complex with *HAdV5* and viral proteins may have been damaged after UV irradiation of **TAL3PYR**-treated cells, the virus was still able to enter cells and complete its infectious cycle.

## 5. Conclusions

In this study, we designed and synthesized tripodal tripyrene-polyamine **TAL3PYR**, characterised by 5+ net positive charge at biorelevant conditions (pH 7.0) and excellent solubility and stability in aqueous medium. Detailed spectrophotometric characterisation of **TAL3PYR** pointed to extensive intramolecular pyrene-stacking in aqueous medium and DMSO, characterised by strong hypochromic and bathochromic changes in UV spectrum, as well as by pyrene excimer emission at about 500 nm. At variance to aqueous or DMSO solution, **TAL3PYR** exhibited mostly unstacked conformation in EtOH.

The novel **TAL3PYR** was strongly but non-selectively bound to grooves of various ds-DNA or ds-RNA by µM binding constants. Moreover, the addition of **TAL3PYR** strongly affected the secondary structure of all ds-DNA/RNA, suggesting extensive unwinding and loss of chirality of double-stranded helices. Such a structural collapse was in line with polynucleotide backbone negative charge compensation by strongly positive **TAL3PYR**, as noted for previously studied **L1** or **L2** analogues (Scheme 1). Although **TAL3PYR** and previously studied analogue **L1** (Scheme 1) have a similar net positive charge (+5 to +6), the **TAL3PYR** intramolecular aggregation of pyrenes hampered fully efficient electrostatic interaction with negatively charged DNA-backbone, resulting in a decreased affinity toward ds-DNA/RNA by order of magnitude. However, **BFTA**-analogue (Scheme 1), characterised by two large aromatic units and only 3+ positive charge, showed only one order of magnitude lower affinity with respect to **TAL3PYR**, demonstrating that aromatic interactions with DNA can compensate lower positive charge.

The **TAL3PYR** efficiently cleaved supercoiled DNA upon irradiation as a consequence of the yrene-mediated production of singlet oxygen. The **TAL3PYR** negligible toxicity in vitro against A549 cell line upon light irradiation changed, reaching measurable bioactivity at submicromolar concentrations. Further experiments showed that light irradiation increased total ROS production in cells treated with **TAL3PYR**, which resulted in typical markers for ROS-induced cellular damage, namely cell swelling and the augmentation of cellular complexity.

As **TAL3PYR** induced plasmid DNA cleavage upon irradiation, we believe that increased ROS production could be caused by increased mitochondrial DNA damage. Namely, increased mitochondrial ROS production has been reported in human airway epithelial cells exposed to benzo(a)pyrene [56]. Similarly, exposure to benzo(a)pyrene has been shown to cause mitochondrial dysfunction and injury to neural cells [57].

Regardless of the fact that we observed strong complex between **TAL3PYR** and *HAdV5*, this had no influence on virus infectivity. It is possible that in *HAdV5*, where the DNA is protected by a protein coat, **TAL3PYR** successfully binds to virus proteins, but after exposure to UV radiation it does not sufficiently damage the proteins responsible for virus infectivity, i.e., it does not cause antiviral activity in this setting.

Thus, **TAL3PYR**, owing to strong interactions with ds-DNA/RNA as well as by photoinduced bio-bioactivity, could be considered a promising lead compound for two-photon-excited PDT applications [24].

## Figures and Tables

**Table 1:** Electronic absorption and emission data of **TAL3PYR** and referent **PyrBZ** at various solvents: EtOH, DMSO, pH 7, sodium cacodylate buffer, *I* = 0.05 M.

Solvent	*ε*/M^−1^ cm^−1^	^a^ *Φ_f_*	^b^ λ_exc_/nm	^b^ λ_em_/nm	τ/ns ^b^	χ^2^
EtOH (400 nm)		0.71 ± 0.05	340	400	5.54 (62%) 22.29 (38%)	1.094
EtOH (486 nm)			340	486	4.22 (−B ^d^) 44.76 (−B ^d^)	1.032
DMSO		^c^	340	486	41.00 (76%) 59.19 (24%)	1.093
buffer	3.88 ± 0.11× 10^4^	0.13 ± 0.05	340	486	3.84 (−B ^d^) 21.00 (−B ^d^)	1.150
PyrBZ buffer	6.26 ± 0.12 × 10^4^	0.15 ± 0.02	340	400	2.5 (<1%) 100.3 (99.2%)	1.060

^a^ Absolute fluorescence quantum yield was determined with an integrating sphere SC-30, Edinburgh Inst., for Argon purged solutions, by λ_exc_ = 353 nm. Measurements were performed in triplicate and the average values are reported. The associated errors correspond to the maximum absolute deviation. ^b^ In the TC-SPC measurements the solutions were purged with argon, and samples were excited by a pulsing diode at 340 nm. ^c^ Not determined. ^d^ Negative pre-exponential factor (−B) is indicative of the formation of aggregates in the excited state [42,43].

**Table 2:** Thermal denaturation (^a^ ∆*T*_m_*/*°C) of AT-DNA (*c*(AT-DNA) = 2.5 × 10^−5^ M) and poly A-poly U (*c*(RNA) = 2.5 10^−5^ M) at various ratios (r_[(compd)]/[polynucleotide]_) upon the addition of **TAL3PYR**. Performed at pH 7.0 (sodium cacodylate buffer, *I* = 0.05 M).

	r	AT-DNA	pApU
**TAL3PYR**	0.05	22.8	1.7
	0.1	24.7	3.7

^a^ Error in Δ*T*_m_-values ± 0.5 °C.

**Table 3:** Binding constants (log*K_s_*) ^a^ of **TAL3PYR** with ds-polynucleotides calculated by various methods, performed at pH = 7.0, sodium cacodylate buffer, *I* = 0.05 M.

Compound	Method	AT-DNA	GC-DNA	AU-RNA	ct-DNA
TAL3PYR	CD titration	6.4	6.4	6.5	6.0 (6) ^b^
TAL3PYR	^c^ IDA_50%_	6.4	6.0	6.3	6.5
^d^ L1	^d^ ITC	^d^ 7.0	^d^ 8.2	^d^ 7.1	-
^e^ BFTA	^e^ Fluorimetric titration	-	-	^e^ 5.0	^e^ 5.8

^a^ Processing of titration data by means of Scatchard equation [38] gave values of the ratio *n* [bound dye]/[polynucleotide] = 0.1 and 0.3. For easier comparison, all log *K_s_* values were re-calculated for fixed *n* = 0.2. Correlation coefficients were >0.99 for all calculated values of log *K_s_*. ^b^ Estimated from UV/Vis titration, Supplementary Information Figure S4; ^c^ Estimated log *K_s_* = log (IDA_50%_ × 1 × 10^6^ M^−1^), which is *K_s_* of EtBr with ds-DNA [37]. ^d^ Scheme 1, published results [18]; ^e^ Scheme 1, published results [19].

## Data Availability

Not applicable.

## Supplementary Materials

The following supporting information can be downloaded at: https://www.mdpi.com/article/10.3390/pharmaceutics15092197/s1, Figure S1: ^1^H NMR spectrum of TAL3PYR in DMSO:D_2_O 3:1. Note: some ethanol is present in the sample; Figure S2. ^13^C NMR spectrum of TAL3PYR in DMSO:D_2_O 3:1. Note: some ethanol is present in the sample; Figure S3. HSCQ NMR spectrum of TAL3PYR in DMSO:D_2_O 3:1; Figure S4. (a) UV/Vis spectra changes of TAL3PYR at different concentrations (concentration range from 5 × 10^−6^–2 × 10^−5^ M); (b) Dependence of Abs different λ_max_ on *c*(TAL3PYR), at pH = 7, sodium cacodylate buffer, *I* = 0.05 M; Figure S5. Changes of the UV/Vis spectra of TAL3PYR with temperature increase and upon cooling back to 25 °C (temperature range from 25–95 °C) at pH = 7, sodium cacodylate buffer, *I* = 0.05 M; Figure S6. Changes of emission spectra with increase of temperature, *c*(TAL3PYR) = 2.5 and 5 × 10^−7^ M at λ_exc_ = 351 nm, at pH = 7, Na cacodylate buffer, *I* = 0.05 M; Figure S7. Comparison of experimental fluorescence decay traces of Tal3PYR under argon (*c*(Tal3PYR) = 5.0 × 10^−6^ M; in sodium cacodylate buffer, *I* = 0.05 M, pH = 7) at λ_exc_ = 351 nm and λ_em_ = 400 and 486 nm; Figure S8. (a) Changes in UV/Vis spectrum of TAL3PYR (*c* = 1.0 × 10^−6^ M) upon titration with ctDNA (*c* = 1 × 10^−7^–1.2 × 10^−5^ M); (b) Dependence of TAL3PYR absorbance at λ_max_ = 348 nm on c(ctDNA), at pH 7.0, sodium cacodylate buffer, *I* = 0.05 M; Figure S9. CD titration of ct-DNA (*c* = 3.0 × 10^−5^ M) with TAL3PYR at molar ratios *r* = [TAL3PYR]/[polynucleotide] at pH = 7.0, buffer sodium cacodylate, *I* = 0.05 M; Table S1: Electronic absorption data of TAL3PYR and PYR; Table S2. Groove widths and depths for selected nucleic acid conformation.
