# Supplementary material for: Novel Tripodal Polyamine Tris-Pyrene: DNA/RNA Binding and Photodynamic Antiproliferative Activity
Source: Pharmaceutics. 2023 Aug 25;15(9):2197. doi: 10.3390/pharmaceutics15092197 (PMC10536304; doi:10.3390/pharmaceutics15092197)
Supplement: Supplementary file 1 [file pharmaceutics-15-02197-s001.zip › pharmaceutics-2532595-supplementary.pdf]

Supporting information

Novel Tripodal Polyamine Tris-Pyrene: DNA/RNA Binding and Photodynamic Antiproliferative Activity

Marta Jurković [1], Marijana Radić Stojković [1], Ksenija Božinović [2], Davor Nestić [2], Dragomira Majhen [2], Estefanía Delgado-Pinar [3], Mario Inclán [3,4], Enrique García-España [3,*] and Ivo Piantanida [1,*]

1 Division of Organic Chemistry and Biochemistry, Ruđer Bošković Institute, Bijenička Cesta 54, 10000 Zagreb, Croatia; marta.koscak@irb.hr (M.J.); mradic@irb.hr (M.R.S.)
2 Division of Molecular Biology, Ruđer Bošković Institute, Bijenička Cesta 54, 10000 Zagreb, Croatia;
ksenija.bozinovic@irb.hr (K.B.); davor.nestic@irb.hr (D.N.); dragomira.majhen@irb.hr (D.M.)
3 Department of Inorganic Chemistry, Institute for Molecular Science, University of Valencia, Catedratico Jose Beltran 2, 46980 Paterna, Spain; estefania.delgado@uv.es (E.D.-P.); mario.inclan@uv.es (M.I.)
4 Escuela Superior de Ingeniería, Ciencia y Tecnología, Universidad Internacional de Valencia (VIU), 46002 Valencia, Spain
* Correspondence: enrique.garcia-es@uv.es (E.G.-E.); pianta@irb.hr (I.P.)

Contents

Synthesis 2
Chemico-physical properties 5
Interactions with DNA/RNA 8

1. Synthesis

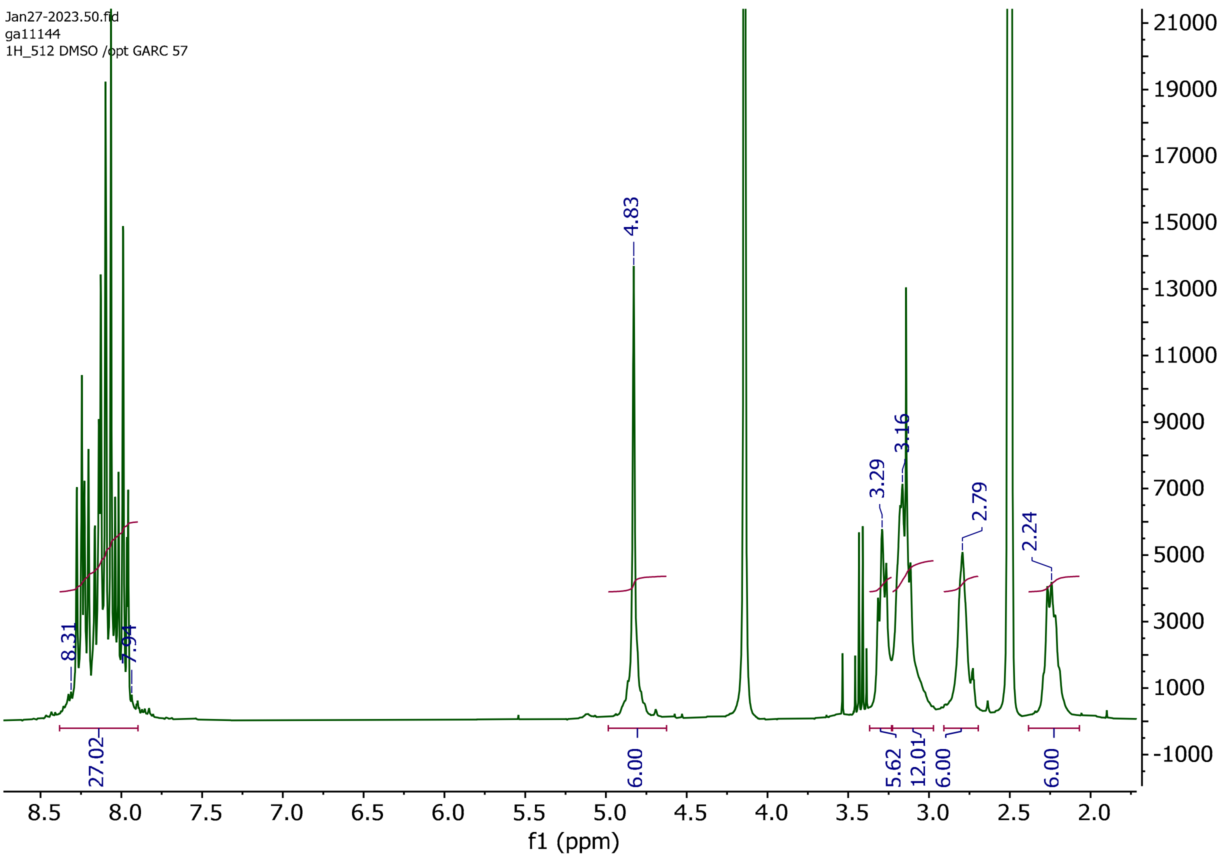

Figure S1. ^{1}H NMR spectrum of **TAL3PYR** in DMSO:D_2O 3:1. Note: some ethanol is present in the sample.

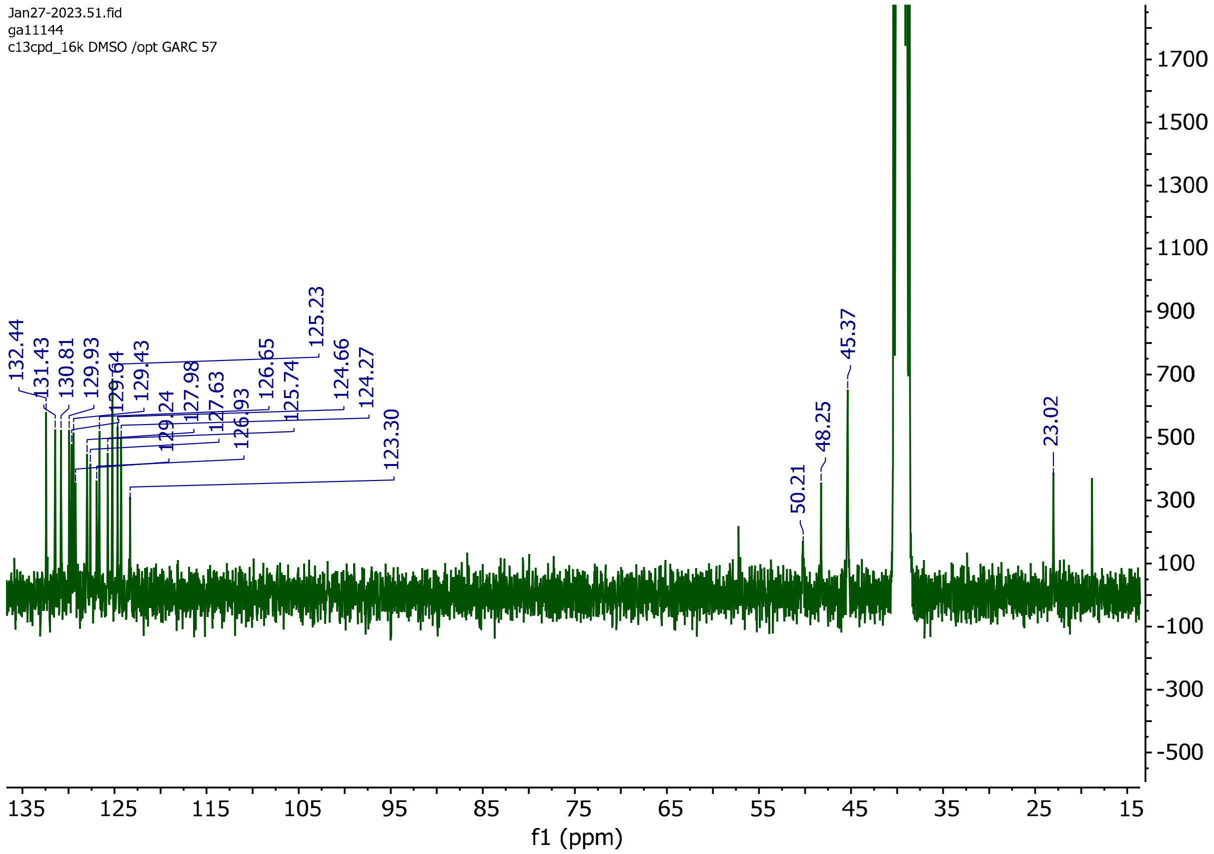

Figure S2. ^{13}C NMR spectrum of **TAL3PYR** in DMSO:D_2O 3:1. Note: some ethanol is present in the sample.

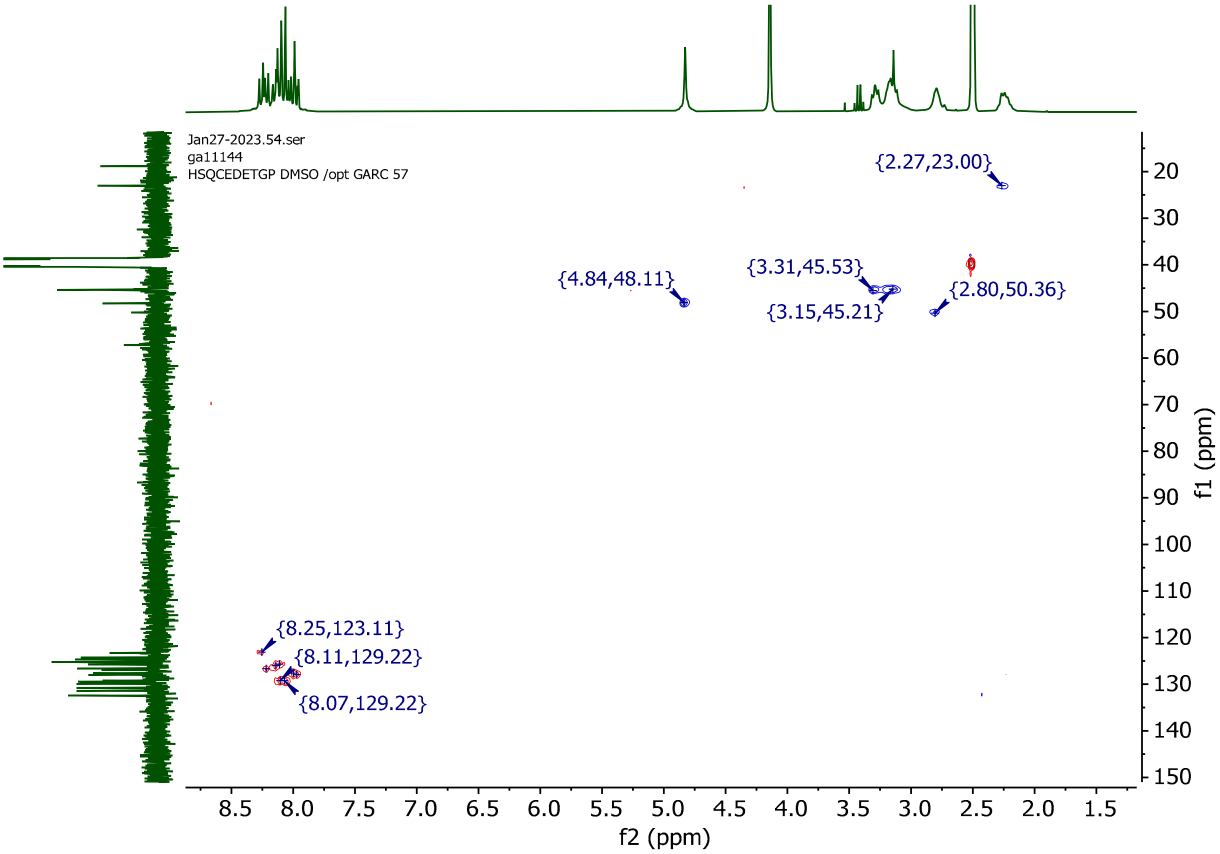

Figure S3. HSCQ NMR spectrum of **TAL3PYR** in DMSO:D_2O 3:1.

2. Chemico-physical properties

Table S1. Electronic absorption data of **TAL3PYR** and **PYR**.

	λ_{max} / nm	$\varepsilon \times 10^3$ / mmol^{-1} cm^2
**TAL3PYR**	236	75.6
	280	57.6
	334	42.9
	351	38.8
**PYR**	276	62.1
	326	41.5
	342	59.7

[a] Sodium cacodylate buffer, *I* = 0,05 M, pH = 7,0.

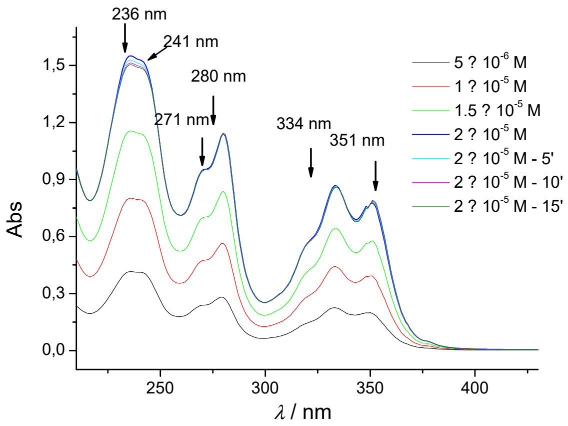

(**a**)

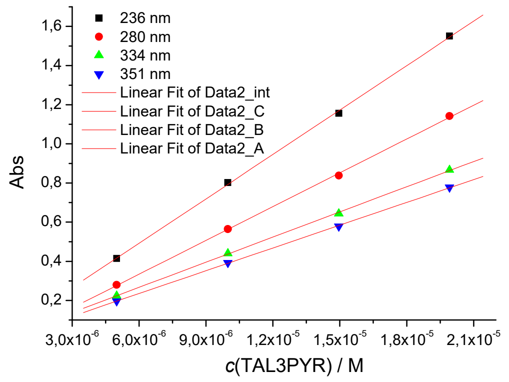

(**b**)

Figure S4. a) UV/Vis spectra changes of **TAL3PYR** at different concentrations (concentration range from 5 × 10^{-6}- 2 × 10^{-5} M); **b**) Dependence of Abs different λ_{max} on *c*(**TAL3PYR**), at pH=7, sodium cacodylate buffer, *I*=0.05 M.

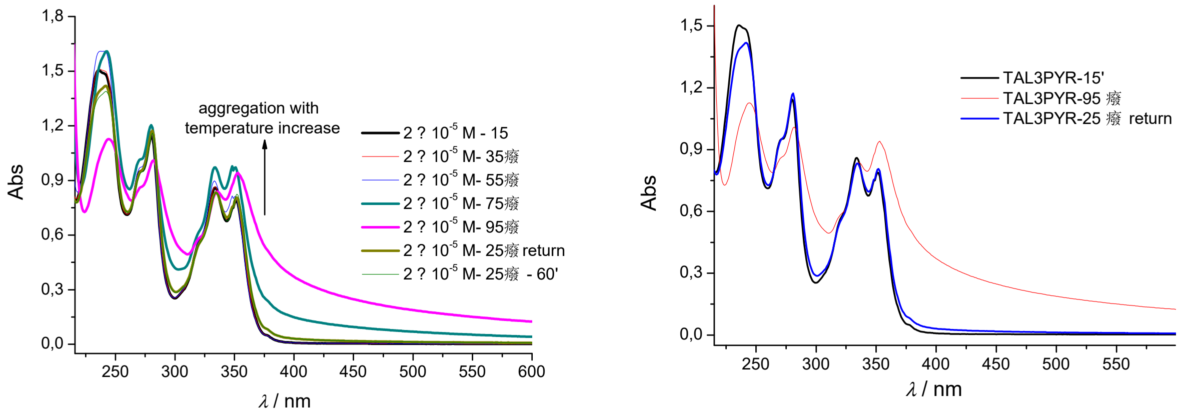

(a) **(b)**

Figure S5. Changes of the UV/Vis spectra of **TAL3PYR** with temperature increase and upon cooling back to 25 °C (temperature range from 25 - 95 °C) at pH=7, sodium cacodylate buffer, *I*=0.05 M.

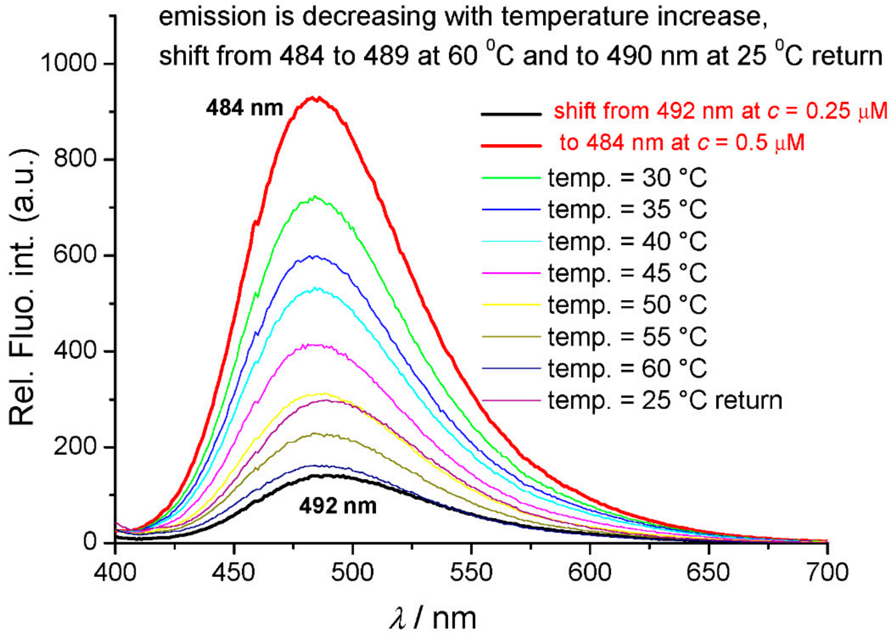

Figure S6. Changes of emission spectra with increase of temperature, *c*(**TAL3PYR**)= 2.5 and 5 × 10^{-7} M at λ_{exc}= 351 nm, at pH=7, Na cacodylate buffer, *I*=0.05 M.

TC-SPC (Time Correlated Single Photon Counting) measurements were performed on an Edinburgh FS5 spectrometer equipped with a pulsed LED at 340 nm. The duration of the pulse was ≈ 1 μs. Fluorescence signals at 400 and 486 nm were monitored over 1023 channels with the time increment of ≈20 ps/channel. The decays were collected until they reached 3000 counts in the peak channel. A suspension of silica gel in H_2O was used as a scattering solution to obtain instrument response function (IRF). Prior to the measurements, the solutions were purged with a stream of argon for 20 min. The measurement was performed at rt (25 °C). Decays of fluorescence were fit to a sum of exponentials according to equation (Fit: A+B1exp(-t/t1)+B2exp(-t/t2)+B3exp(-t/t3)).

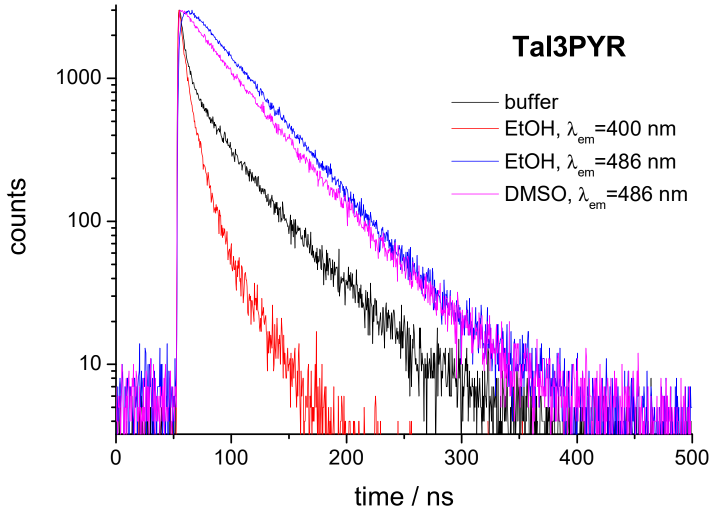

Figure S7. Comparison of experimental fluorescence decay traces of **Tal3PYR** under argon (c(Tal3PYR) = 5.0 × 10^{-6} M; in sodium cacodylate buffer, I=0.05 M, pH=7) at λ_{exc} = 351 nm and λ_{em} = 400 and 486 nm. Fitting results were obtained by reconvolution fit.

3. Interactions with DNA/RNA:

Table S2. Groove widths and depths for selected nucleic acid conformation [1,2].

Structure type	Groove width [Å]		Groove depth [Å]	
	major	minor	major	minor
[a] poly rA – poly rU	3.8	10.9	13.5	2.8
[b] ct-DNA (48% of GC-pairs)	11.4	3.3	7.5	7.9
[b] poly dAdT – poly dAdT	11.2	6.3	8.5	7.5
[c] poly dGdC – poly dGdC	13.5	9.5	10.0	7.2

[a] A - helical structure

[b] B - helical structure

[c] B - helical structure with sterically blocked minor groove by amino groups of guanines

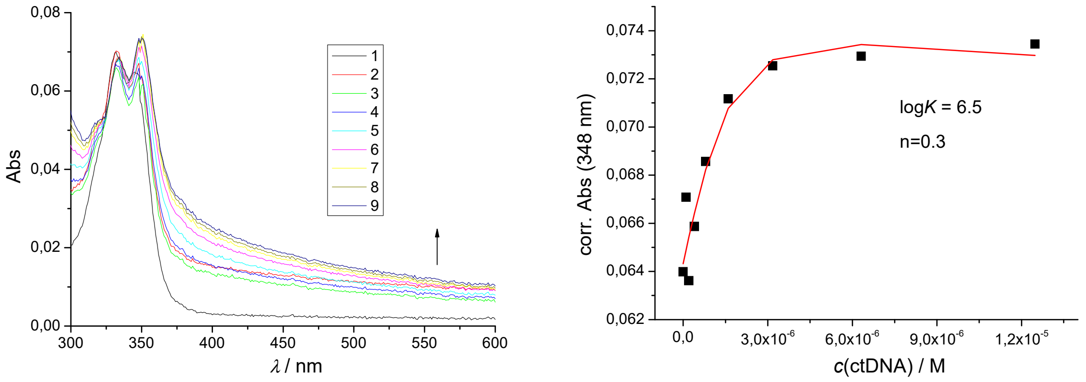

(**a**) (**b**)

Figure S8. a) Changes in UV/Vis spectrum of **TAL3PYR** (c = 1.0 × 10^{-6} M) upon titration with ctDNA (c = 1 × 10^{-7} – 1.2 × 10^{-5} M); **b**) Dependence of **TAL3PYR** absorbance at λ_{max} = 348 nm on c(ctDNA), at pH 7.0, sodium cacodylate buffer, I = 0.05 M.

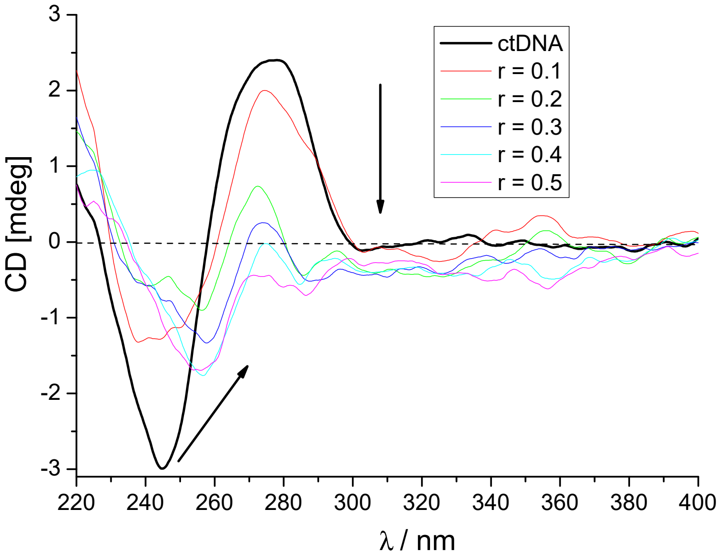

Figure S9. CD titration of ct-DNA (c = 3.0 × 10^{-5} M) with **TAL3PYR** at molar ratios r = [**TAL3PYR**] / [polynucleotide] at pH = 7.0, buffer sodium cacodylate, I = 0.05 M.

---

1. Parish, J.H. Principles of Nucleic Acid Structure: By W Saenger. pp 556. Springer-Verlag, New York. 1984. ISBN 3-540-90761-0. Biochem. Educ. 1985, 13, 92.
2. Cantor, C.R.; Schimmel; P.R. Biophysical Chemistry, WH Freeman and Co.: San Francisco, USA, 1980; pp. 1109-1181.
